# Propolis as a Potential Therapeutic Agent to Counteract Age-Related Changes in Cartilage: An In Vivo Study

**DOI:** 10.3390/ijms241814272

**Published:** 2023-09-19

**Authors:** Consuelo Arias, Bélgica Vásquez, Luis A. Salazar

**Affiliations:** 1 Escuela de Kinesiología, Facultad de Odontología y Ciencias de la Rehabilitación, Universidad San Sebastián, Santiago 8380000, Chile; consuelo.arias@gmail.com; 2 Department of Basic Sciences, Faculty of Medicine, Universidad de La Frontera, Avenida Francisco Salazar 01145, Temuco 4811230, Chile; 3 Centre of Excellence in Morphological and Surgical Studies, Universidad de La Frontera, Avenida Francisco Salazar 01145, Temuco 4811230, Chile; 4 Center of Molecular Biology and Pharmacogenetics, Scientific and Technological Bioresource Nucleus, Universidad de La Frontera, Avenida Francisco Salazar 01145, Temuco 4811230, Chile

**Keywords:** osteoarthritis, propolis, aging

## Abstract

Aging is intricately linked to chronic low-grade systemic inflammation, which plays a significant role in various age-related conditions, including osteoarthritis (OA). The aging process significantly influences the development of OA due to alterations in cartilage composition, reduced proteoglycan content, dysregulation of growth factor signaling, and heightened oxidative stress. Propolis, a natural product renowned for its potent antioxidant and anti-inflammatory properties, has the potential to mitigate age-induced changes in cartilage. The primary objective of this study was to rigorously assess the impact of in vivo propolis treatment on the histopathological characteristics of knee articular cartilage in senescent rats. This study involved a cohort of twenty male Sprague–Dawley rats, randomly allocated into four distinct groups for comparative analysis: YR (control group consisting of young rats), SR (senescent rats), SR-EEP (senescent rats treated with an ethanolic extract of propolis, EEP), and SR-V (senescent rats administered with a control vehicle). This study employed comprehensive histological and stereological analyses of knee articular cartilage. Propolis treatment exhibited a significant capacity to alleviate the severity of osteoarthritis, enhance the structural integrity of cartilage, and augment chondrocyte density. These promising findings underscore the potential of propolis as a compelling therapeutic agent to counteract age-related alterations in cartilage and, importantly, to potentially forestall the onset of osteoarthritis.

## 1. Introduction

Aging is a multifaceted process characterized by chronic low-grade systemic inflammation, leading to progressive structural and functional loss in various bodily systems. This chronic inflammation disrupts the balance between inflammatory and anti-inflammatory pathways, contributing significantly to the development of age-related conditions, including neurodegenerative diseases, atherosclerosis, metabolic syndrome, and musculoskeletal disorders like osteoarthritis (OA) [1]. The etiology of OA involves a multifactorial interplay of risk factors, including age, female gender, obesity, anatomical factors, muscle weakness, joint injury, mechanical stress, and abnormal joint mechanics [2].

Notably, OA is particularly influenced by the aging process and standing as one of its most critical risk factors for its development [3,4,5,6]. Aging-related alterations in cartilage composition such as decreased expression of COL2A1 (a marker of healthy cartilage) and increased expression of COL1A1 (a marker of OA) have been observed [7,8,9]. Furthermore, reduced proteoglycan content alters cartilage’s biomechanical properties, leading to increased stiffness and susceptibility to fatigue failure due to heightened biomechanical stress [10,11,12]. Age-related declines in growth factor signaling and increased oxidative stress further link aging to OA pathogenesis [13,14].

The impact of aging extends beyond the cartilage itself, affecting synovial homeostasis, resulting in a decline in synthetic chondrocyte activity and progressive thinning of articular cartilage [10,11]. This gradual loss of chondrocyte ability to maintain and restore articular cartilage [15,16] heightens the risk of age-related degeneration and fibrillation of the articular surface [4,10]. As aging and OA progress, there is an altered expression of various proinflammatory cytokines and transcription factors [4,7,17], exacerbating the degradative phenotype and leading to a significant decrease in chondrocyte cell density [4,6,7,8,11]. Additionally, homeostatic mechanisms like autophagy become compromised in aging cartilage, correlating with cell death and OA development [8,18].

Despite the prevalence of OA and its detrimental impact on individuals’ quality of life, no cure is currently available, and some existing drugs are associated with significant adverse effects [19,20,21]. Consequently, there is an urgent need to explore therapeutic alternatives targeting age-induced changes in cartilage underlying OA development.

Natural products, such as propolis, have garnered attention due to their potential beneficial effects, including antioxidant and anti-inflammatory properties that combat chronic inflammation associated with aging [22,23]. Propolis, a resinous substance rich in polyphenols collected and transformed by bees, exhibits multiple pharmacological properties. These properties encompass hepatoprotective, antioxidant, and anti-inflammatory effects [23,24,25]. Furthermore, propolis has demonstrated its ability to modulate the immune system effectively in vivo [26,27,28,29]. 

In the context of cartilage health, propolis extract has exhibited remarkable protective properties, partly attributed to its scavenger action. These findings suggest its potential application in combination with synthetic drugs for the treatment of joint diseases [30]. Moreover, studies have observed that treatment with aqueous propolis in rats with radiation-induced arthritis significantly outperforms diclofenac, effectively preventing cartilage breakdown and inhibiting the increase in nitric oxide (NO) [31]. Additionally, propolis has shown promise in improving mechanical hyperalgesia and gait patterns in an induced osteoarthritis (OA) model, primarily due to its anti-inflammatory and antioxidant properties [32]. Treatment with propolis in chondrocytes stimulated with IL-1β has demonstrated beneficial effects, including reduced production of NO, decreased expression of MMP13, and increased expression of COL2A1, indicating its potential anti-inflammatory effects in vitro [33]. Furthermore, oral propolis treatment has been reported to mitigate cartilage degeneration in rats with experimentally induced OA [34]. Propolis has also exhibited a positive role in bone tissue repair, enhancing bone formation in rats with bone defects and increasing the number of chondrocytes and osteoblasts in a rat model of femur fracture [35,36]. Additionally, there is evidence suggesting that propolis and its various components may possess anti-aging effects and potential benefits for chronic diseases. For example, in skin health, propolis has been linked to anti-aging effects through the direct inhibition of PI3K activity [37]. Its antioxidant and anti-inflammatory properties make it a potential candidate for ameliorating the symptoms of metabolic syndrome and associated chronic diseases [38,39]. Many of these effects of propolis are likely attributed to synergistic interactions among its various components [35]. 

In conclusion, age-related changes in cartilage play a crucial role in the pathogenesis of osteoarthritis (OA). Disruptions in cartilage homeostasis, alterations in inflammatory pathways, and reduced chondrocyte activity significantly contribute to the development of this debilitating condition. In this context, the exploration of natural products such as propolis holds great promise as an intervention to counteract age-related changes and prevent the onset of OA. The objective of this study was to evaluate the effect of in vivo treatment with propolis on the histopathological characteristics of knee articular cartilage in senescent rats.

## 2. Results

### 2.1. In Vivo Treatment with EEP Does Not Generate Significant Changes in the Survival of the Animals

**Figure 1:**
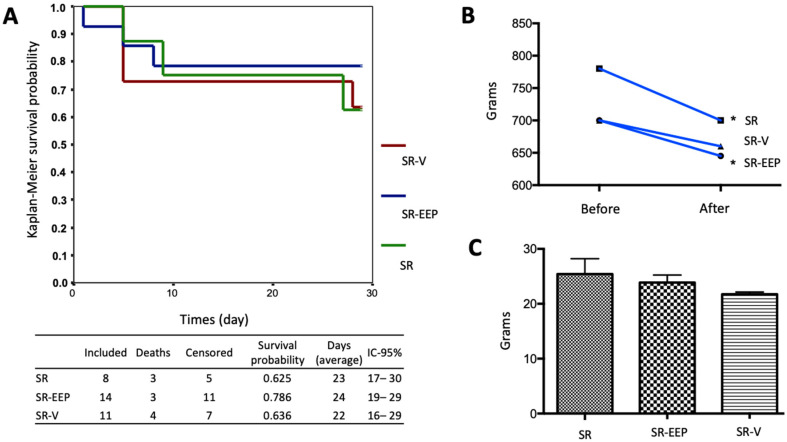
Animal survival analysis. (**A**) Kaplan–Meier survival curve for SRs, SR-EEPs, and SR-Vs. IBM SPSS statistics for Windows, version 22.0, released 2013, Armonk, NY, USA. IBM Corp., was used. (**B**) Body weight variation before and after oral gavage treatment in senescent rats in the different groups. (**C**) Variation in liver weight in relation to oral gavage treatment in senescent rats in the different groups. GraphPad Prism version 5.00, GraphPad Software, San Diego, CA, USA, was used in both analyses. One-way ANOVA, Dunn’s multiple comparison test. *t* test. * = *p* < 0.05. SR: Senescent Rats; SR-EEP: Senescent Rats treated with EEP; SR-V: Senescent Rats treated with vehicle.

The effect of oral treatment with EEP on the survival of senescent animals was investigated using a Kaplan–Meier survival curve. However, the analysis did not reveal significant differences in survival among all the groups (Figure 1A). To assess potential effects of EEP treatment on the animals, body weight variation before and after treatment was examined and compared between the groups. Notably, a significant decrease in body weight was observed in the SR and SR-EEP groups, while no significant differences were found in the SR-V group. Nevertheless, when comparing these variations between the groups, no significant changes were observed (Figure 1B). Additionally, liver weight was analyzed to evaluate possible drug toxicity. However, no significant differences in liver weight were observed between the different groups (Figure 1C).

### 2.2. Descriptive Histological Analysis of Knee Articular Cartilage Suggests Key Differences between Young and Senescent Animals

**Figure 2:**
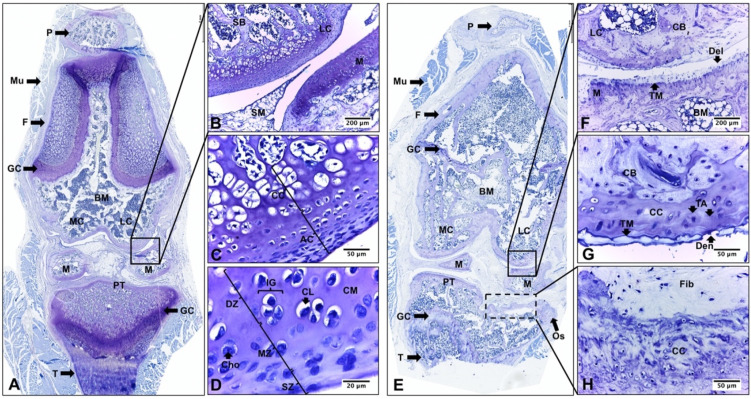
Histopathological comparisons of knee joint of young and senescent rats. Toloudine blue staining of 3-week- and 25-month-old rat knee was performed. YR: knee joint of young rat (**A**–**D**); SR: knee joint of senescent rat (**E**–**H**); P: patella; Mu: muscle; F: femur; GC: growth cartilage; BM: bone marrow; AC: articular cartilage; LC: lateral condyle; MC: medial condyle; M: meniscus; TP: tibial plateau; T: tibial; SM: synovia membrane; SZ: superficial zone; MZ: mid zone; DZ: deep zone; TM: tidemark; CC: calcified cartilage; CB: compact bone; SB: spongy bone; Cho: chondrocytes; CL: cartilage lacuna; CM: cartilage matrix; IG: isogenous group; TA: tidemark advancement; Os: osteophytes; Fib: fibrocartilage; Del: delamination (focal superficial zone matrix loss extending to MZ); Den: denudation (matrix loss extending to calcified cartilage interface); dotted line area: articular bone plate deformation.

The histological analysis of knee articular cartilage in young rat knees (YR group) revealed a well-organized structure with three distinct zones (superficial, middle, and deep). The chondrocytes exhibited proper orientation and arrangement, and no morphological alterations or proliferative changes were observed. The calcified cartilage presented hypertrophic/apoptotic chondrocytes surrounded by a calcified matrix (Figure 2A–D). In stark contrast, senescent rat knees (SR group) exhibited significant degenerative changes. These changes encompassed reduced cartilage thickness, architectural alterations affecting the three zones, chondrocyte death, and matrix depletion. Notably, the tidemark was distinctly visible. Furthermore, characteristic histological features of osteoarthritis (OA) were evident, signifying a degradation process in the articular cartilage. In the medial structures of the joint, superficial abrasion and delamination extended into the MZ. In contrast, the lateral structures displayed even more pronounced degenerative changes, featuring areas of erosion and denudation of the articular cartilage. Additionally, abnormal bone remodeling with sclerotic changes in the subchondral bone was observed. It is noteworthy to mention the presence of peripheral osteophytes in the PT region (Figure 2E–H, Table 1).

### 2.3. Descriptive Histological Analysis of Knee Articular Cartilage Suggests Beneficial Effects of In Vivo EEP Treatment

**Figure 3:**
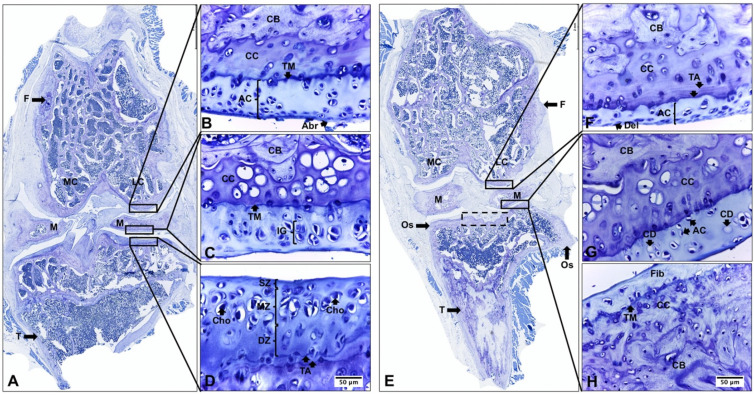
Histopathological comparisons of knee joint of senescent rats with and without propolis treatment. SR-EEP: knee joint of senescent rat treated with propolis (**A**–**D**). SR-V: knee joint of senescent rat treated with vehicle (**E**–**H**). F: femur; AC: articular cartilage; LC: lateral condyle; MC: medial condyle; M: meniscus; T: tibial; SZ: superficial zone; MZ: mid zone; DZ: deep zone; TM: tidemark; CC: calcified cartilage; CB: compact bone; Cho: chondrocytes; AC: apoptotic chondrocyte; DC: dead chondrocyte; IG: isogenic group; TA: tidemark advancement; Os: osteophytes; Fib: fibrocartilage; Abr: abrasion (focal loss of surface portion of superficial zone); Del: delamination (focal superficial zone matrix loss extending to MZ); dotted line area: articular bone plate deformation. Toluidine blue stain.

In the SR-EEP group, articular cartilage of the knee presented a more organized appearance with increased thickness, articular surface, and matrix cationic staining compared to the SR and SR-V groups. The superficial zone appeared smoother and more regular, although some abrasion of the superficial layer was observed in specific zones. The middle and deep zones showed a prominent proliferation of chondrocytes, which were arranged in isolation or forming isogenic groups. The majority of chondrocytes were in their lacunae and displayed a hypertrophic appearance. The tidemark was evident, and its duplication was observed in FC, M, and TP. The calcified cartilage exhibited increased toluidine blue staining. Additionally, some necrotic hypertrophic chondrocytes were observed (Figure 3A–D). The articular cartilage and subchondral bone of the knee in the senescent rat, of the SR-V group, exhibited histological characteristics similar to the SR group (Figure 3E–H).

### 2.4. In Vivo EEP Treatment Reduces Osteoarthritis Severity in Knee Articular Cartilage

**Figure 4:**
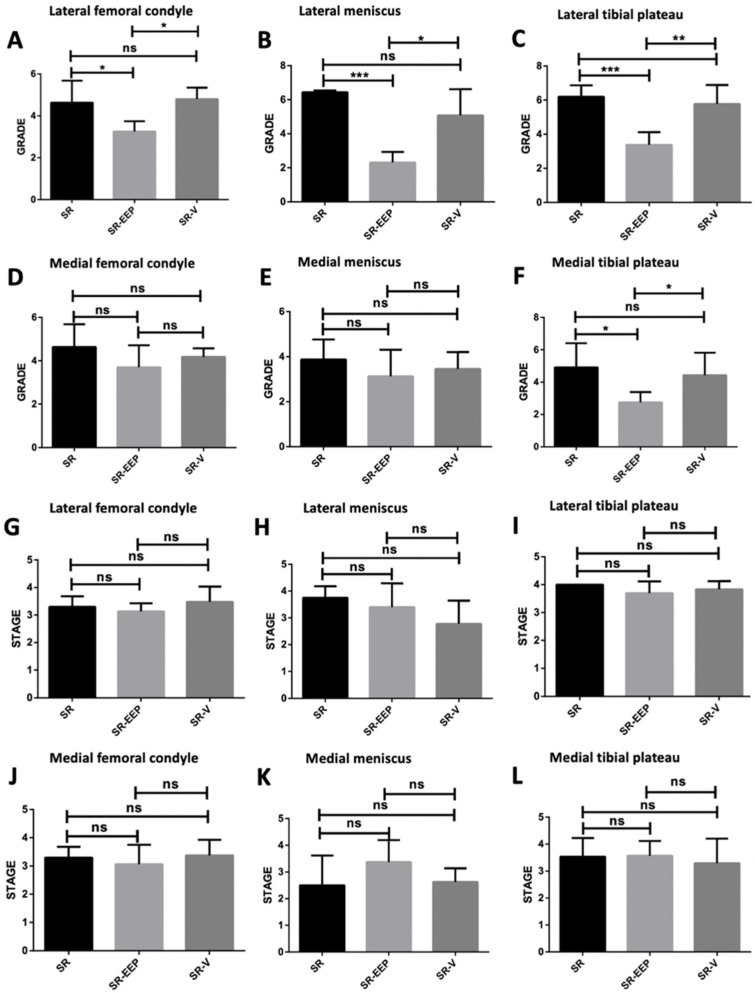
Classification of knee articular cartilage damage was performed according to scores obtained from the OARSI scale. SR: knee joint of senescent rat. SR-EEP: knee joint of senescent rat treated with propolis. SR-V: knee joint of senescent rat treated with vehicle. Grade of damage of articular cartilage of (**A**) lateral femoral condyle, (**B**) lateral meniscus, (**C**) lateral tibial plateau, (**D**) medial femoral condyle, (**E**) medial meniscus, (**F**) medial tibial plateau. Stage of damage of articular cartilage of (**G**) lateral femoral condyle, (**H**) lateral meniscus, (**I**) lateral tibial plateau, (**J**) medial femoral condyle, (**K**) medial meniscus, (**L**) medial tibial plateau. GraphPad Prism version 5.00, *t* test. * *p* < 0.05; ** *p* < 0.005; *** *p* < 0.0005; ns; not significant; *n* = 5.

OARSI scoring (Osteoarthritis Research Society International) of the knee joints revealed that both the lateral M and TP exhibited the highest degree of osteoarthritis in the SR group (Figure 4C,F). Notably, the SR-EEP group showed a significant reduction in osteoarthritis severity in the articular cartilage of FC, M, lateral TP, and medial TP compared to the SR and SR-V groups (Figure 4A–C,F). The SR-V group did not exhibit significant differences in osteoarthritis severity compared to the SR group in all analyzed structures (Figure 4A–F). While there were no significant differences in the stage of osteoarthritis between the groups in the structures analyzed, the lateral TP in all groups showed the highest horizontal extent of cartilage damage (Figure 4G–I).

These findings suggest that in vivo treatment with EEP had beneficial effects on knee articular cartilage in senescent rats, reducing osteoarthritis severity and promoting a more organized cartilage structure.

### 2.5. Stereological Analysis Revealed That Propolis Treatment Significantly Reduces Chondrocyte Depletion Caused by Aging

**Figure 5:**
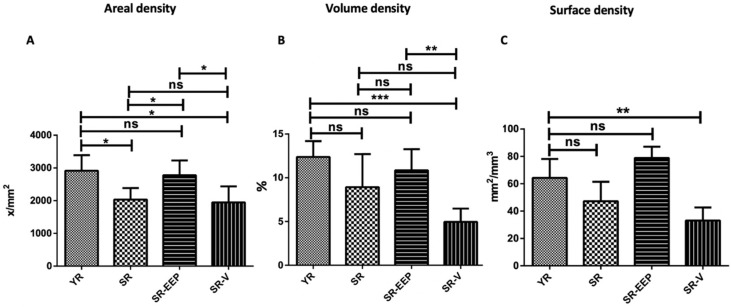
Stereological analysis of knee articular cartilage. YR: knee joint of young rat. SR: knee joint of senescent rat. SR-EEP: knee joint of senescent rat treated with propolis. SR-V: knee joint of senescent rat treated with vehicle. (**A**) areal density, (**B**) volume density, (**C**) surface density. Stereological analysis performed with the Stepanizer program. GraphPad Prism version 5.00, One-way ANOVA, *t* test. * *p* < 0.05; ** *p* < 0.005; *** *p* < 0.0005; ns; not significant; *n* = 5.

The density of chondrocytes per area (NA) was significantly higher in the SR-EEP group compared to both the SR and SR-V groups, and it did not differ significantly from the YR group (Figure 5A). In addition, the percentage of chondrocytes in the articular cartilage (VV) was significantly higher in the SR-EEP group compared to the SR-V group (Figure 5B). Both the volume density and surface density (SV) of the SR-EEP group did not show significant differences with the YR group (Figure 5B,C).

## 3. Discussion

As life expectancy continues to rise, the importance of finding strategies that promote healthy aging becomes paramount. Aging is intricately linked with a gradual loss of physiological integrity and cellular homeostasis, resulting in a decline in cellular function over time. These age-related changes, potentially driven by chondrocyte senescence, represent a common molecular mechanism underlying both age-associated and posttraumatic osteoarthritis (OA), and they substantially contribute to the development of various aging-related diseases, including OA [40,41,42]. 

It is essential to emphasize that aging and osteoarthritis (OA) are distinct yet interconnected processes. While advanced age stands as the primary risk factor for OA, it is crucial to recognize that not all elderly individuals develop this condition. This suggests that OA is not an inevitable consequence of aging and may not be its sole cause [43]. This distinction is exemplified by juvenile osteoarthritis, which occurs in young individuals, highlighting that OA can manifest independently of the natural aging process. The relationship between OA and aging is indeed intricate, encompassing changes in cartilage structure and cellular processes over time. However, it is crucial to note that OA is not solely responsible for its development; rather, it is age-related changes that lay the foundation for OA development [44]. 

The present study offers provides valuable insights into the morphological alterations observed in the knee articular cartilage of senescent rats. Histological analysis reveals significant degenerative changes, including reduced cartilage thickness, altered architecture of the cartilage zones, chondrocyte death, and matrix depletion (Figure 2 and Figure 4, Table 1). Furthermore, characteristic histological features indicative of OA was identified, signifying a degradation process in the articular cartilage. These features encompassed surface abrasion and delamination in the less affected medial structures, as well as erosion and denudation of articular cartilage in the more affected lateral joint striations. Additionally, sclerotic changes in the subchondral bone and peripheral osteophytes were observed in the PT region (Figure 2 and Figure 4, Table 1). These observed changes were quantitatively assessed using the OARSI scale, which revealed a higher degree of OA in the senescent group of rats (Figure 4).

Moreover, the histological analysis highlights the potential beneficial effects of in vivo treatment with ethanolic extract of propolis (EEP) on knee articular cartilage in senescent rats (Figure 3). The EEP-treated group exhibits a more organized cartilage structure with increased thickness and reduced osteoarthritis severity (Figure 3). Stereological analysis further supports these findings, suggesting that EEP treatment significantly reduced chondrocyte depletion caused by aging (Figure 5). Importantly, the study demonstrates that EEP treatment is well-tolerated by the animals and does not induce significant toxicity or adverse effects on the liver (Figure 1). The expected mortality rate is consistent with that published for the age range used [45]. This suggests that EEP at the administered dose should not be toxic and supports the potential application of propolis in diseases related to aging in an older adult population characterized by multiple comorbidities and the need for safer therapeutic options. These results align with previous studies in animals with induced osteoarthritis [34].

In conclusion, this study significantly contributes to our understanding of the potential benefits of propolis treatment on knee articular cartilage in senescent rats, offering a novel approach for managing OA in the aging population. The observed reduction in chondrocyte depletion, improved cartilage structure, and diminished osteoarthritis severity are promising indicators of propolis’ efficacy as a therapeutic intervention. Nevertheless, further research is imperative to fully elucidate the mechanisms of action and safety profile of propolis treatment, paving the way for its potential application in managing OA and promoting healthy aging. In conclusion, this study significantly contributes to our understanding of the potential benefits of propolis treatment on knee articular cartilage in senescent rats, offering a novel approach for managing OA in the aging population. The observed reduction in chondrocyte depletion, improved cartilage structure, and diminished osteoarthritis severity are promising indicators of propolis’ efficacy as a therapeutic intervention. Nevertheless, further research is imperative to fully elucidate the mechanisms of action and safety profile of propolis treatment, paving the way for its potential application in managing OA and promoting healthy aging.

There is substantial scientific evidence regarding the beneficial effects of certain polyphenols on age-related diseases. One of the central contributors to the chronic inflammation associated with aging is the accumulation of senescent cells in aging tissues [46]. Consequently, interventions targeting chronic inflammation could play a pivotal role in addressing age-related diseases. It is worth noting that while selective COX-2 inhibitors have been associated with a higher cardiovascular risk, nonselective COX inhibitors are linked to elevated gastrointestinal risk [47]. Therefore, the pursuit of natural treatments with fewer side effects becomes of paramount importance.

Several natural treatments with anti-inflammatory properties have been proposed as potential anti-aging agents. Curcumin, for instance, exhibits antioxidant characteristics and holds promise in targeting major signaling cascades involved in aging [48]. Additionally, studies have reported that artichoke leaf extract possesses chondroprotective properties in an inflammatory context, significantly reducing MMP13 and PGE2 production while rescuing GAG production [49]. Resveratrol, another extensively studied polyphenol, has demonstrated potential in preventing high-fat diet-induced muscle atrophy in aged rats by reversing mitochondrial dysfunction and oxidative stress, contributing to its anti-aging effects [50]. It has also been proposed for use as a dietary supplement in generating antioxidant and anti-inflammatory effects in healthy individuals [51]. Furthermore, it might help prevent age-related oculopathy partly through the enhancement of mitochondrial function [52].

Propolis has also emerged as a potential therapeutic agent against age-related disorders. Various studies have reported high antioxidant capacities of propolis from different origins, suggesting its potential as a therapeutic candidate in aging-related diseases [53]. Some studies have proposed that ethanol extract of propolis may reduce the production of proinflammatory cytokines in aged mice with induced candidiasis [54]. Additionally, the administration of Brazilian green propolis has shown a positive effect on innate and adaptive immunity in aged mice [55]. In elderly individuals living at high altitudes, ingestion of propolis for over 12 months protected against cognitive decline after reducing systemic inflammation [56]. Propolis has also demonstrated anti-aging potential by avoiding premature senescent phenotypes in yeast [57]. It is worth mentioning that Portuguese propolis may exhibit anti-inflammatory effects in vitro, similar to those obtained from aspirin [32]. Some studies even suggest a synergistic effect of the various compounds found in propolis, reducing oxidative stress and influencing glucose and cholesterol plasma levels associated with aging [58]. Since 2009, the Italian Society of Natural Medicine has declared propolis to be a safe product to use [59,60]. Furthermore, other studies on the long-term administration of propolis have demonstrated that it is nontoxic and safe for use in senescent animals and older people [56,58]. These findings strongly support its potential application in age-related diseases among an older population characterized by pharmacological iatrogenesis and multiple comorbidities.

EEP (ethanol extract of propolis), as a complex mixture of various polyphenols, may owe its observed effects to the synergy between multiple compounds [33,61]. It is important to acknowledge that while this study lacks biochemical analyses that could explain the key metabolites responsible for propolis’ beneficial effects on cartilage, it represents an initial step that adds value to the physiological relevance determined through animal studies. Nevertheless, it is essential to recognize that despite the considerable potential of natural treatments due to their broad pharmacological effects, clinical studies supporting these effects remain limited.

However, it is crucial to emphasize that while EEP may have beneficial effects on the severity of osteoarthritis (OA), this disease is multifactorial and complex. The limitations in EEP’s capacity to modify the horizontal extent of cartilage damage may be attributed to several factors affecting its efficacy. To address these limitations and enhance EEP’s potential as a therapeutic agent in OA, several research guidelines should be considered:

The accessibility and distribution of the compound may be limited in specific areas of the joint, impacting its ability to fully modify the extent of cartilage damage. The dose and treatment duration could influence its effectiveness. If the dose is insufficient or the treatment duration is inadequate, achieving a significant modification in cartilage damage extent may be challenging. While these animal studies provide important insights into the suggested effect of oral propolis, bioavailability analyses are required to determine which final components are found in the plasma and then whether the components are reflected in the synovial fluid. On the other hand, it is not clear how well the findings translate to humans, due to potential differences in nutrient bioavailability and metabolism [62]. Evaluating different forms of propolis formulations that enhance accessibility, improve the bioavailability of propolis and its bioactive compounds, and distribution in the joint should be explored. Specific formulations could enable sustained and targeted release, maximizing therapeutic effects.

Preclinical studies in animal models that more accurately replicate human osteoarthritis at different stages should be conducted. Whether certain subgroups of patients may benefit more from propolis treatment based on genetic factors, biomarkers, or specific disease characteristics should also be investigated. Personalizing the treatment may improve efficacy and reduce response variability. In addition, it is essential to incorporate a study group to account for histological changes in senescent articular cartilage prior to treatment (24 months), so that future research can better assess the potential effects of EEP.

Comprehensive studies should be carried out to understand the mechanisms of action of propolis in osteoarthritis, and to explore how it interacts with inflammatory and antioxidant pathways, as well as other pathological routes involved in the disease. This research can identify the stages of the disease where propolis may be most effective and how to enhance its action in specific cartilage areas. The feasibility of combining propolis with other therapeutic agents commonly used in osteoarthritis treatment, such as anti-inflammatories, analgesics, or regenerative therapies needs investigating. Combining different treatments may synergize their effects and lead to improved outcomes.

The long-term safety of propolis treatment should be evaluated to ensure there are no significant adverse effects associated with prolonged use, as well as the determination of the optimal propolis dosage for osteoarthritis treatment, potentially through dose–response studies to identify the most effective amount without undesirable side effects. Although some studies indicate that propolis may have an effect on CYP450 enzymes, in humans, the effect is negligible, and its use is supported by the large number of bioactivities it possesses [63]. Additionally, it is essential to address the limitation related to the number of animals used per condition in this study. Future projects should consider increasing the number of animals per condition for improved generalizability of results.

Currently, treatment options for osteoarthritis primarily focus on managing symptoms and slowing disease progression [64,65]. Despite the widespread use of certain medications like glucosamine and cortisone, their efficacy in significantly regenerating damaged articular cartilage remains limited [66]. Consequently, there is a growing need for innovative treatments targeting the underlying cellular pathology of osteoarthritis. Recent advancements in cartilage regeneration strategies have explored potential therapeutic interventions to restore native cartilage and delay osteoarthritis progression [67,68]. Given these considerations, further research is warranted to explore the potential effects of this natural compound on various pathologies associated with aging.

## 4. Materials and Methods

### 4.1. Characterization of Ethanolic Extract of Propolis (EEP)

The crude propolis was obtained from a mountainous area near the town of Cunco, La Araucanía, Chile (latitude −38°58′40.46″, longitude −72°1′15.73″). Its processing and characterization were conducted according to the methodology described in our laboratory [31]. The polyphenol present in the highest amount was pinocembrin, determined to be 44 mg/mL (44 mg mL^−1^) [31].

### 4.2. Animals

Experimental procedures were conducted in accordance with the *Guide for the Care and Use of Laboratory Animals* [69] and the experimental protocol was approved by the Scientific Ethical Committee of the Universidad de La Frontera, Chile (Protocol Number 015/2016). Twenty male Sprague–Dawley rats were housed in a temperature-controlled environment with 12 h light/dark cycles, where they received food and water *ad libitum*. The animals were randomly separated into four groups of five individuals per group (Figure S1): YR (control group of young rats, 3 weeks old), SR (group of 25-month-old senescent rats), SR-EEP (group of 24-month-old senescent rats treated with ethanolic extract of propolis, EEP, at 200 mg/kg/day), and SR-V (group of 24-month-old senescent rats administered only the vehicle, same volume of EEP according to weight). The dose was selected according to previous studies [34,70]. Both EEP and the vehicle were administered daily for one month with a previous 4 h fast. The animals in group YR were euthanized after 3 weeks, while the remaining animals were euthanized at 25 months of age. Euthanasia was performed using an overdose of ketamine (160 mg/kg) and xylazine (20 mg/kg, Figure S1). The determination of sample size was based on the bioethical principles of Russell and Burch’s 3Rs for animal experimentation: replacement, reduction, and refinement (Russell and Burch, 1959). Our study was based on Cruz-Orive and Weibel’s premise that five cases per group represent a sample that allows for a statistically significant result. In general, in histology, quantitative methods are based on discrete random values, which often fall into a binomial distribution that is the basis of the binomial test of statistical significance; thus, the probability is calculated as *p* = (1/2)^n^, where n is the number of cases in the sample. Thus, with *n* = 5, a *p* = 0.03 is obtained, below the statistical level normally used as statistically significant [71,72].

### 4.3. Histological Processing

After euthanasia of the animals, one knee joint was randomly obtained from each animal. For preparation of coronal sections, joints were flexed at 120 degrees and fixed in 10% buffered formalin (1.27 mol/L formaldehyde in 0.1 M phosphate buffer pH 7.2) for 48 h, then decalcified in 10% buffered EDTA for 90 days. Subsequently, the samples were dehydrated in ascending alcohols, rinsed in xylene, and embedded in Paraplast (Fisher Scientific, Waltham, MA, USA). Using a Leica^®^ RM 2255 microtome, 5 µm thick sections were cut at 200 µm intervals and mounted on slides (Superfrost plus) [73]. To optimally evaluate the joint, successive sections of the deepest planes of the joint were stained with toluidine blue, visualized with an optical microscope (Leica^®^ DM 2000 LED), and photographed with a Leica^®^ MC 170 HD digital camera. Then, only one slice per joint was selected, considering the plane of the blockage that crosses the lesion to the greatest extent and that presents the most pronounced alterations [48]. These sections were visualized and scanned using the TissueFAXS i PLUS Cytometer TissueGnostics Axio Observer 7 Carl Zeiss GmbH System (TissueGnostics GmbH, Vienna, Austria).

### 4.4. Histological Analysis

**Figure 6:**
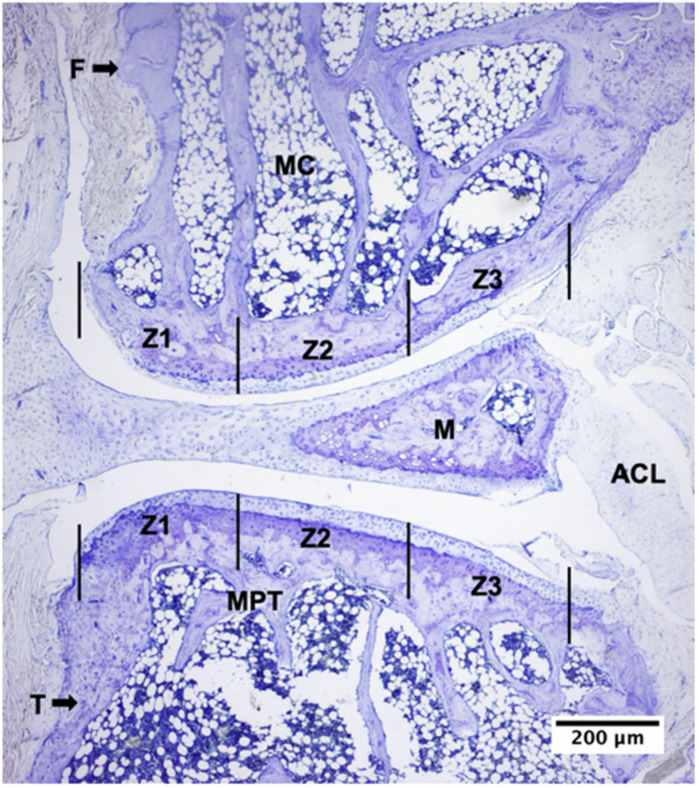
Knee joint of senescent rat. Toloudine blue staining of the 25-month-old rat knee was performed. F: femur; MC: medial condyle; M: meniscus; T: tibial; MTP: medial tibial plateau; ACL: anterior cruciate ligament; Z1: zone 1; Z2: zone 2; Z3: zone 3.

A descriptive analysis of the femoral condyles (FC), menisci (M), and tibial plateau (TP) was performed. The cartilage was described from superficial to deep, starting with the superficial zone (SZ), followed by the middle zone (MZ), the deep zone (DZ), calcified cartilage, articular bone plate, and subchondral trabecular bone. The OARSI histopathological evaluation system for osteoarthritic cartilage was applied independently by the authors BV and CA in each sample [73]. The scale classifies the different grades of cartilage, where grade 0 corresponds to normal tissue and grades 1 to 6 are relative to OA. Grades 1 to 4 OA involve changes in the cartilage only, while in grades 5 and 6, the subchondral bone is also included. The OA stratification method defines four stages, based on the horizontal extent of the affected cartilage surface, regardless of the degree of underlying OA. The cartilages of the FC, M, and TP were evaluated separately on each slide in each group [74,75,76]. The FC and TP, medial and lateral, were divided into three zones of equal width using an ocular micrometer [74]. Zone 1 corresponds to the segment adjacent to the synovium at the lateral margin of the joint; zone 2 corresponds to the intermediate segment; and zone 3 corresponds to the segment adjacent to the cruciate ligaments (Figure 6).

### 4.5. Stereological Analysis

For the stereological study of chondrocytes, the articular cartilage of the medial and lateral femoral condyles (FC), menisci (M), and tibial plateau (TP) was analyzed. Five fields per structure were observed in each histological section [51]. The slides were visualized under a Leica^®^ DM2000 LED stereological microscope and photographed with a Leica^®^ MC170 HD digital camera. The 36-point test system designed by STEPanizer^®^ was utilized, and the following parameters were determined: areal density (N_A_), volume density (V_V_), and surface area density (S_V_) of the chondrocytes. The N_A_ was quantified using the equation N_A_ = Q/A_T_, where Q represents the number of observations in a given area considering the forbidden lines, and A_T_ is the total area of the test system. V_V_ was estimated using the formula: V_V_ = P_P_/P_T_ (100%), where P_P_ represents the number of points touching the chondrocytes and P_T_ is the total number of points in the system. S_V_ was determined according to the following equation S_V_ = (2 × I)/L_T_, where I represents the number of intersections touching the structure, and L_T_ is the total length of the lines of the 36-point test system.

### 4.6. Statistical Analysis

A descriptive statistical analysis was performed by calculating the mean of the scores obtained on the OARSI scale for each group. The Mann–Whitney U test was used to compare the groups, and a *p*-value less than 0.05 was considered statistically significant. The analyses were conducted using the STATA 15.1 program and GraphPad Prism version 5.00.

## Figures and Tables

**Table 1:** Histological characteristics of articular cartilage and subchondral bone of the femoral condyles, meniscus and tibial plateau in knees of young and senescent rats. YR: control group, knees of young rats (3 weeks old); SR: senescent group, knees of senescent rats (25 months old); SZ: superficial zone; MZ: mid zone; DZ: deep zone; CC: calcified cartilage; SB: subchondral bone.

Structure	YR	SR
SZ	The surface is smooth and continuous. It presents small and flat or round chondrocytes, which are aligned parallel to the collagen fibers and the surface.	Irregular and discontinuity surface. Depending on the severity of OA and laterality of the joint structure, abrasion or focal delamination, erosion or denudation with a greater stage of involvement is observed.
MZ	It consists of undifferentiated cells and spherical chondrocytes that are grouped together and surrounded by a proteoglycan matrix (chondrons).	Presence of reactive (clustered with loss of chondron orientation) or dead chondrocytes. Depletion of matrix staining and focal rarefaction.
DZ	It presents round and larger chondrocytes, organized in isogenic groups. Deep hypertrophic chondrocytes are observed.	It presents similar characteristics to MZ. The borderline between MZ and DZ are not very evident due to the lesser thickness of the cartilage and loss of articular cartilage.
Tidemark	Absent	Present. Progress and duplication of the tidemark is observed.
CC	Chondrocytes are hypertrophic/apoptotic. Calcified cartilage matrix with traces of bony trabeculae and vascular infiltration.	Lower cellularity, with presence of dead and/or apoptotic chondrocytes. Calcified cartilage matrix.
SB	Subchondral bone of trabecular type. The bone marrow is dominated by blood elements.	Sclerotic bone. Thicker articular bone plate (bone/marrow ratio). Presence of reparative fibrocartilage. Thin trabeculae and wider medullary spaces, with predominance of yellow bone marrow. Activation of connective tissue at lateral interfaces.
Osteophytes	Absent	Present. They are mainly observed in TP.

## Data Availability

Data are contained within this article. Raw data and materials are available from the authors upon reasonable request.

## Supplementary Materials

The following supporting information can be downloaded at: https://www.mdpi.com/article/10.3390/ijms241814272/s1.
