# Supplementary material for: Propolis as a Potential Therapeutic Agent to Counteract Age-Related Changes in Cartilage: An In Vivo Study
Source: Int J Mol Sci. 2023 Sep 19;24(18):14272. doi: 10.3390/ijms241814272 (PMC10532056; doi:10.3390/ijms241814272)
Supplement: Supplementary file 1 [file ijms-24-14272-s001.zip › ijms-2570448-supplementary.pdf]

Supplementary Materials

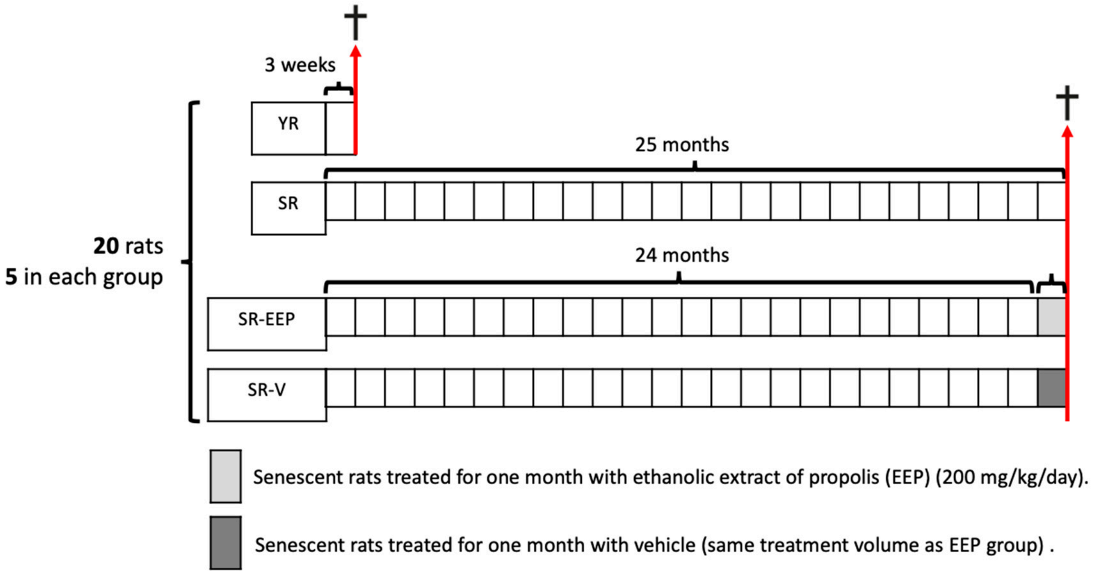

Figures S1. **Experimental procedure of the study.** Twenty male Sprague–Dawley rats were housed in a temperature-controlled environment with 12 h light/dark cycles, where they received food and water *ad libitum*. The animals were randomly separated into four groups of five individuals per group: YR (control group of young rats, 3 weeks old), SR (group of 25-month-old senescent rats), SR-EEP (group of 24-month-old senescent rats treated with ethanolic extract of propolis, EEP, at 200 mg/kg/day), and SR-V (group of 24-month-old senescent rats administered only the vehicle, same volume of EEP according to weight).
